# Classical acceleration temperature (CAT) in a box

**DOI:** 10.1038/s41598-024-72890-2

**Published:** 2024-10-03

**Authors:** Ahsan Mujtaba, Maksat Temirkhan, Yen Chin Ong, Michael R. R. Good

**Affiliations:** 1 Physics Department & Energetic Cosmos Laboratory, Nazarbayev University, 010000 Astana, Qazaqstan Kazakhstan; 2 Physics Department, NED University of Engineering & Technology, Karachi, 75270 Pakistan; 3 Physics Department, Lahore University of Management Sciences (LUMS), Lahore, 54792 Pakistan; 4 Center for Gravitation and Cosmology, Yangzhou University, Yangzhou, 225002 China; 5 Shanghai Frontier Science Center for Gravitational Wave Detection, Shanghai Jiao Tong University, Shanghai, 200240 China; 6 Leung Center for Cosmology and Particle Astrophysics, National Taiwan University, Taipei, 10617 Taiwan

**Keywords:** Statistical physics, thermodynamics and nonlinear dynamics, Statistical physics, Thermodynamics, Physics, Quantum physics, Theoretical physics

## Abstract

A confined, non-relativistic, accelerating electron is shown to emit thermal radiation. Since laboratories face spatial constraints when dealing with rectilinear motion, focusing on a finite total travel distance combines the benefits of simple theoretical analysis with prospects for table-top experimentation. We demonstrate an accelerated moving charge along an asymptotically static worldline with fixed transit distance and non-relativistic maximum speed, emitting self-consistent analytic power, spectra, and energy. The classical radiation is Planck distributed with an associated acceleration temperature. This is the first fully parametrized, spectrum-solved, finite-distance worldline.

## Introduction

Quantum radiation from thermal black holes^1^ is studied in analog by quantum radiation from moving mirrors^2–4^. Ongoing studies exploit the similarities between these systems in investigating quantum radiation^5–9^. Interestingly, classical radiation from a moving point charge has a one-to-one map to the quantum radiation from a moving mirror, see e.g., Ref^10–12^. Recently, the accelerating electron from beta decay has been experimentally shown to emit classical thermal radiation commensurate with infinite particle production^13–15^.

A completely evaporated black hole should release a finite amount of particles (IR-finite)^16^. A moving mirror must return to rest for finite particle emission. It is a strong constraint on the motion to return to rest. So it is no surprise that rectilinear motions possessing asymptotic rest with solved Bogolyubov coefficients have provided insight into the general classical-quantum character of particle creation and acceleration radiation^12,16–20,20–23^. Perhaps the primary benefits to exploring these trajectories have been the finite energy emission and finite particle production. Nevertheless, asymptotically resting worldlines also suffer no information loss (unitary) and are free from IR divergences.

Asymptotic rest pays the cost of quantum purity^21,24^ and is a physically well-motivated way to preserve the unitary interaction between the mirror and its quantum field^25^. In the context of the semi-classical flying mirror (the mirror is classical with a position and velocity simultaneously known while the fields are quantized), information loss occurs when a global acceleration horizon is formed. With asymptotic rest, late-time light rays will never evade the mirror and cannot descend past a horizon into its analog black hole.

Of course, quite independently of its utility as a tool to model Hawking evaporation of black holes, moving mirror solutions are interesting in and of themselves. Finding analytic solutions exhibiting certain properties one wishes to study is generally difficult. If we want to move beyond theoretical toy models to laboratory experiments, we would like to construct a solution whose trajectory is bounded in space since a laboratory is finite in size. The problem is that, in the literature, only one asymptotically static mirror is known to travel a finite distance; see Table 1. This trajectory (‘Arctx’) cannot be simultaneously solved for its Bogolyubov coefficients and parametrized in terms of maximum speed^26^. Thus, studying how its maximum speed relates to its particle spectrum is challenging. Likewise, it is unknown whether or not finite travel affects particle creation.

Faced with these intractabilities, we approach these issues by investigating a new solution that can be simultaneously solved and parametrized. We introduce the first spectrum-solved finite-distance worldline, expressed in terms of its maximum speed. The trajectory facilitates an examination of confinement on particle spectrum and count. The result is Planck-distributed thermal radiation.

The structure of the paper is as follows. In Sect. Electron in a box, we review the essential dynamics for the trajectory, developing an intuition for the power and the energy emission. Here, we reveal the interesting physics of ‘big-box’ energy production. Section Spectral analysis is devoted to spectral analysis of the radiation, running into intractable generality. Nevertheless, we find a spectrum for a specific box size and demonstrate consistency with the energy emission. With the spectrum in hand, we compute effective temperature functions based on the trajectory dynamics in Sect. Classical acceleration temperature and demonstrate thermal radiation. In Sect. Particle production, we use the spectral results to draw several conclusions about the photon particle production. Section Conclusions summarizes the main findings, emphasizing the radiation is thermal from a finite travel region, i.e., a CAT in the box. Units are $c = \mu _0 = \epsilon _0 =1$, except in temperature results where we have reinstated SI units. The electron charge is given by *e*, and Planck’s constant $\hbar$ appears in all Kelvin temperatures and quantum results.

**Table 1:** There are a handful of asymptotically resting worldlines.

Asymptotic static motions	Distance travelled
Walker-Davies^17^	$\infty$
Arctx^26^	Finite & unparametrized
Self-dual^18^	$\infty$
da Vinci-betaK^19,20^	$\infty$
Schwarzschild–Planck^16,20–23^	$\infty$
Fermi–Dirac^12^	$\infty$
Worldline Eq. (1)	Finite & parametrized

The following list chronologically summarizes the known trajectories possessing asymptotic rest with solved Bogolyubov coefficients. Here, ‘unparametrized’ means the energy production (for Arctx) has not been expressed with a parameter that characterizes the distance traveled. See the parametrized energy, Eq. (9), for worldline Eq. (1).

## Electron in a box

**Fig. 1:**
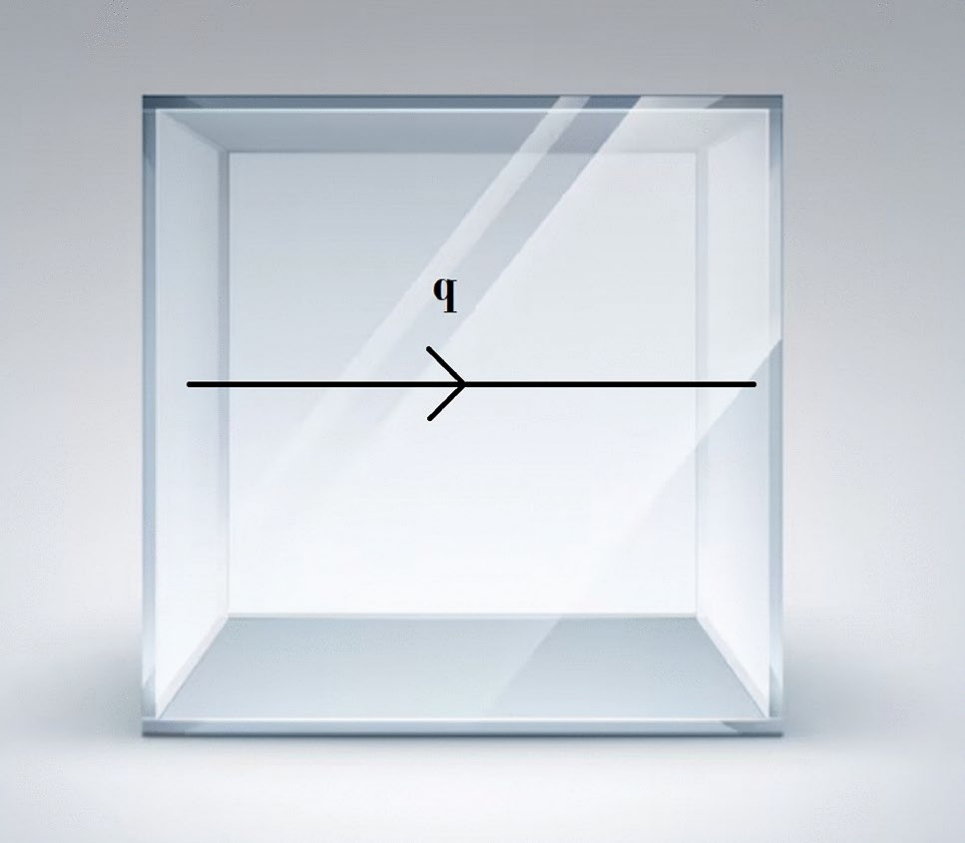
A moving charge, Eq. (1), confined to a geometric cubic volume of space, a ‘box’ with transparent sides (i.e., for our theoretical discussion, it is only a mathematical boundary, not an actual physical box, though the figure is suggestive of possible experimental set-up that would involve actual boundaries). The minimal length of the box is $L = 4 r/\kappa$, where *r* is a dimensionless free parameter, and $\kappa$ is the dimensionful scale of the system with units of acceleration. The charge radiates thermal photons distributed according to a one-dimensional Planck spectrum, Eq. (40), shown explicitly when $r=1$ in the non-relativistic regime. For large box sizes, $r\rightarrow \infty$ (all maximum speeds), thermal radiation is implicit via energy emission, Eq. (12).

We start with an ansatz for the equation of motion of the moving point charge. Assume the charge, such as an electron, travels along a particular worldline confined to a finite space. Allow it to accelerate rectilinearly within this finite distance (confined to a ‘box’; see Fig. 1), where the worldline is:

1 $$\begin{aligned} t(x)=x\left( \frac{1}{s}-\frac{1}{r}\right) +\frac{2}{\kappa }\tanh ^{-1}\left( \frac{\kappa x}{2 r}\right) . \end{aligned}$$

The electron, moving in a straight line, traverses the distance from $x=-2r/\kappa$ to $x=+2r/\kappa$, where *x* is the single space coordinate, $0<s<1$ is the maximum speed of the electron, *t* is the dependent variable and measure of lab time, $\kappa$ is the dimensionful scale of the system with units of acceleration, and $r>0$ is a dimensionless free parameter introduced to characterize displacement conveniently.

**Fig. 2:**
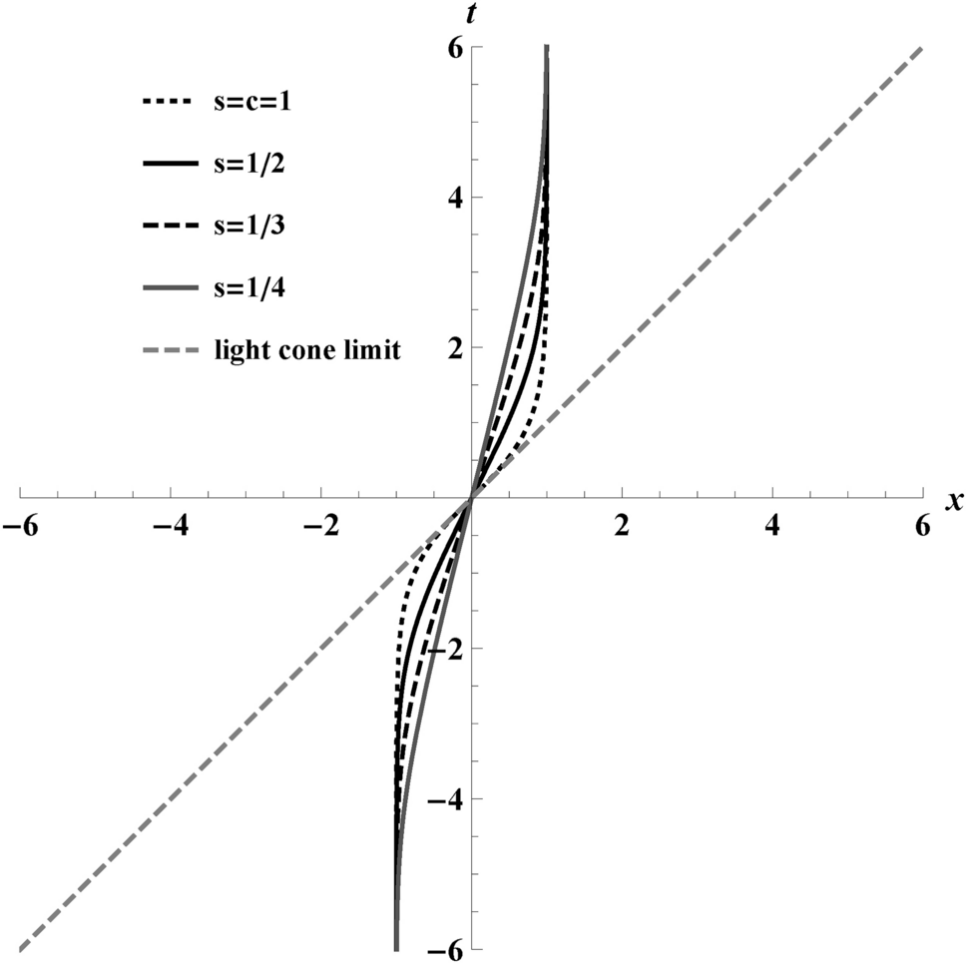
Trajectories, *t*(*x*), from Eq. (1) at $r=1$ with different values of maximum speeds $s\rightarrow [1/4,1/3,1/2,1]$. The dashed gray line in the plot at $45^\circ$specifies the light cone limit, and, by definition, no time-like trajectory can cross it. An electron moves along the trajectory from $x\rightarrow (-1,1)$ at $\kappa =2$ substituted into Eq. (1). Notice the finite distance travelled.

Equation (1) describes a continuous, globally defined, timelike worldline starting from rest, reaching maximum speed *s*, and returning to rest. The key trait is finite distance travel. We have plotted Eq. (1) in Fig. 2 with $\kappa =2$ and $r=1$, depicting $x\rightarrow (-1,1)$. The figure shows the trajectories for different maximum speeds, *s*. These paths travel a finite distance for all values of $0<s<1$. In the causal limit $s\rightarrow 1$, the charge takes the least time to cover the distance.

Let us emphasize that the ‘box’ in Fig. 1 is a fictitious boundary (perfect transparency) rather than a physical boundary (imperfect transparency) typically associated with phenomena like Purcell enhancement in cavity QED^27^. The distinction is important. Purcell enhancement occurs when a radiating particle is confined within a physical cavity, which changes the photon density of states due to the boundaries. This, in turn, leads to an increased spontaneous emission rate for the particle, e.g., Ref^28^. The mode enhancement resulting from these physical boundaries has been proposed to potentially observe the thermality of the Unruh effect in tabletop experiments^29^. In contrast, the ‘box’ we use is a theoretical boundary that demonstrates the 3+1 dimensional context of the problem and the finite distance traveled by the electron along the trajectory, Eq. (1). This boundary is not physical but a geometric cubic volume with transparent sides, demonstrating the charge is confined within a specific region of space. As we will see, the charge within this fictitious three-dimensional boundary radiates thermal photons distributed according to a one-dimensional Planck spectrum.

### Dynamics, *t*(*x*)

The proper acceleration of the electron is useful for understanding the motion and is necessary to calculate the power and total energy emitted. The proper acceleration is, see, e.g., Ref^30^

2 $$\begin{aligned} \alpha (x)=\frac{\!\textrm{d} }{\!\textrm{d}{x}}\gamma (x). \end{aligned}$$

Here the Lorentz factor is $\gamma (x)$=$1/\sqrt{1-({\!\textrm{d}{t}/\!\textrm{d}{x}})^{-2}}$. We obtain $\textrm{d}{t}/\!\textrm{d}{x}$, by taking the derivative of Eq. (1):

3 $$\begin{aligned} \frac{\!\textrm{d}{t}}{\!\textrm{d}{x}}=\frac{1}{s}+\frac{1}{r}\frac{1}{(2r/\kappa x)^2 - 1}. \end{aligned}$$

Inverting Eq. (3), gives the velocity, *v*(*x*), for the trajectory,

4 $$\begin{aligned} v(x)=s\frac{4r^{2}-(\kappa x)^2}{4r^2-(\kappa x)^2(1-s/r)}. \end{aligned}$$

**Fig. 3:**
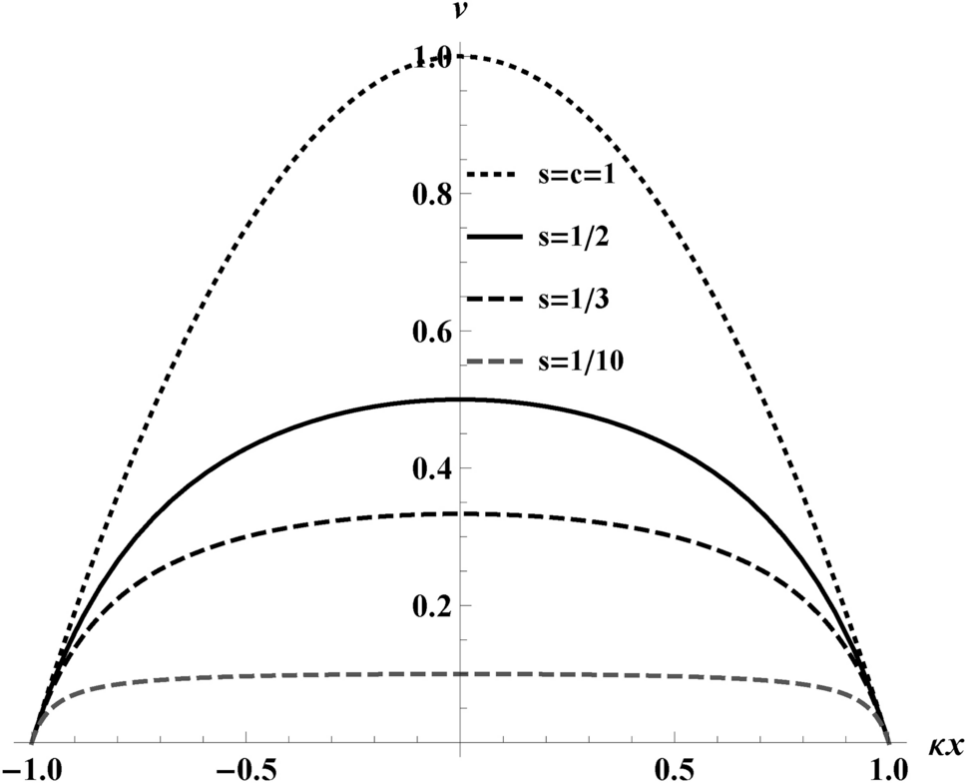
A plot of velocity versus distance (scaled by $\kappa$) traveled, *v* vs $\kappa x$, for different peak velocities, *s*, at $r=0.5$ which ensures the traversal of $\kappa x$ from $(-1,1)$ for visual simplicity, since $\kappa x\rightarrow (-2r,2r)$ depicting the asymptotic resting situation from Eq. (4). The maximum speed is reached at the spatial origin, $v(0) = s$. Notice the smallest maximum speed, $s=0.1$, differs from the smallest maximum speed in Fig. 2. The smaller the maximum speed, the flatter the velocity curve, consistent with an intuition of thermal equilibrium based on time dependence shown by the dashed gray line at $s=1/10$.

Notice when $r \rightarrow \infty$ then $v(x) \rightarrow s$. Notice also that when $s\ll 1$, then $v(x) \approx s$. On plotting Eq. (4) in Fig. 3, we get the expected behavior, which starts from rest $v(-2r/\kappa )=0$, reaches a maximum speed, $v(0) = s$, and comes back to rest $v(+2r/\kappa )=0$ after covering a finite distance.

Expressed as a function of velocity *v*, the proper acceleration $\alpha (v)$ is:

5 $$\begin{aligned} \alpha (v)= \pm \kappa v \gamma ^3 \left( 1-\frac{v}{s}\right) ^{1/2} \left( 1 - \frac{v}{s}+\frac{v}{r}\right) ^{3/2}, \end{aligned}$$

It is also straightforward to use Eq. (4) to find the Lorentz factor and obtain the proper acceleration $\alpha (x)$ from Eq. (2).

**Fig. 4:**
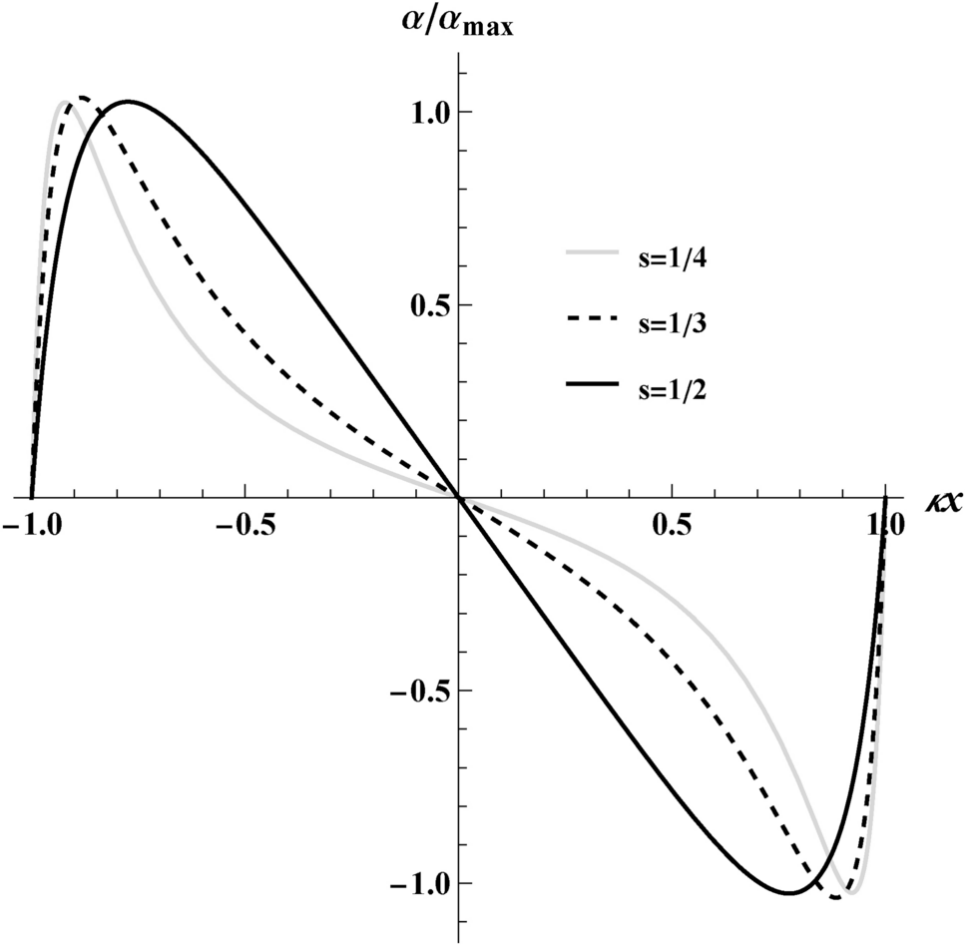
The above is a plot of the proper acceleration, $\alpha (x)$, for various maximum speeds depicting an acceleration increasing on the left side of the box until a maximum and decreasing until $\alpha (0)=0$, and subsequent symmetric deceleration on the right side of the box, confirming the particle to be confined in the box. The accelerations with different maximum speeds are normalized by their maximum accelerations for $s\rightarrow (1/2,1/3,1/4)$ found to be, $\alpha _\text {max}\rightarrow (0.25, 0.13, 0.09)$, respectively.

On plotting $\alpha (x)$ of Eq. (2) in Fig. 4, we see that our electron reaches a maximum speed, *s*, when $\alpha (0)=0$ on the graph. For max speeds, $s \rightarrow 1$, $\alpha \rightarrow \pm \infty$. There, the acceleration and deceleration of the confined electron can be seen for various maximum speeds.

### Larmor power, *P*(*v*)

The power emitted by the electron is given by the Larmor–Liénard relation, parametrized by velocity, *v*, as the independent variable:

6 $$\begin{aligned} P(v)=\frac{e^2}{6\pi }\alpha (v)^2. \end{aligned}$$

With $\alpha (v)^2$ from Eq. (5), the power is an implicit function of *x*, explicitly expressed in terms of velocity, *v*, as:

7 $$\begin{aligned} P(v) = \frac{e^2}{6\pi }\kappa ^2 v^2 \gamma ^6 \left( 1-\frac{v}{s}\right) \left( 1 - \frac{v}{s}+\frac{v}{r}\right) ^{3}. \end{aligned}$$

**Fig. 5:**
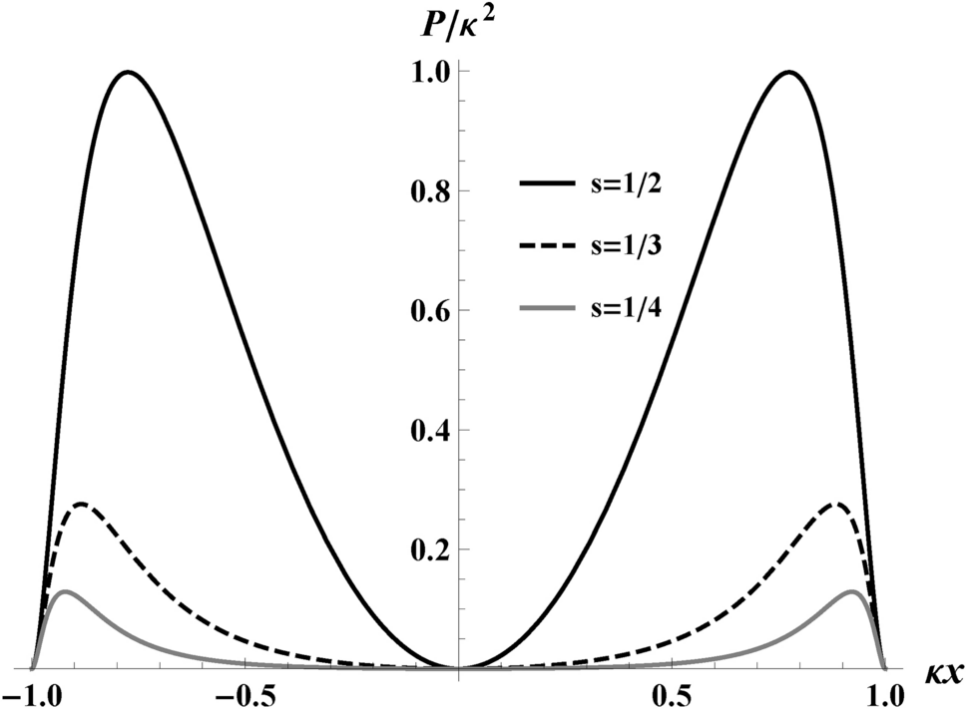
Plots of Larmor power, Eq. (7), as a function of distance, $P/\kappa ^2$ vs $\kappa x$, for different values of maximum electron speeds, $s\rightarrow$[1/2, 1/3, 1/4]. Here, $r=0.5$ to give the distance from the origin as one. For visual simplicity, we have normalized the vertical axis with the maximum power, $P_{\text {max}}/\kappa ^2$, for $s=1/2$. Most of the power is emitted near the edges of the box. As the maximum speed of the massive electron increases to $s\rightarrow 1$, the Lorentz factor in Eq. (7) diverges, $\gamma \rightarrow \infty$, giving rise to an infinite (unphysical) emission of power.

We have plotted the results of the Larmor power as a function of distance *P*(*x*) in Fig. 5, which demonstrates a two-lobe power trend minimizing at $x=0$ where the electron’s maximum speed reaches *s*, then becomes $P = 0$ when the electron comes to rest.

### Energy from power, *E*(*r*,*s*)

We obtain the total finite energy emitted by the electron via integrating the Larmor power, *P*, from Eq. (6) over time. Using the appropriate Jacobian to change the integration variable from $t\rightarrow x$, we confine the electron to its integration bounds:

8 $$\begin{aligned} E=\frac{e^2}{6\pi }\int \limits _{-2r/\kappa }^{+2r/\kappa }\alpha (x)^{2}\frac{dt}{dx}dx. \end{aligned}$$

**Fig. 6:**
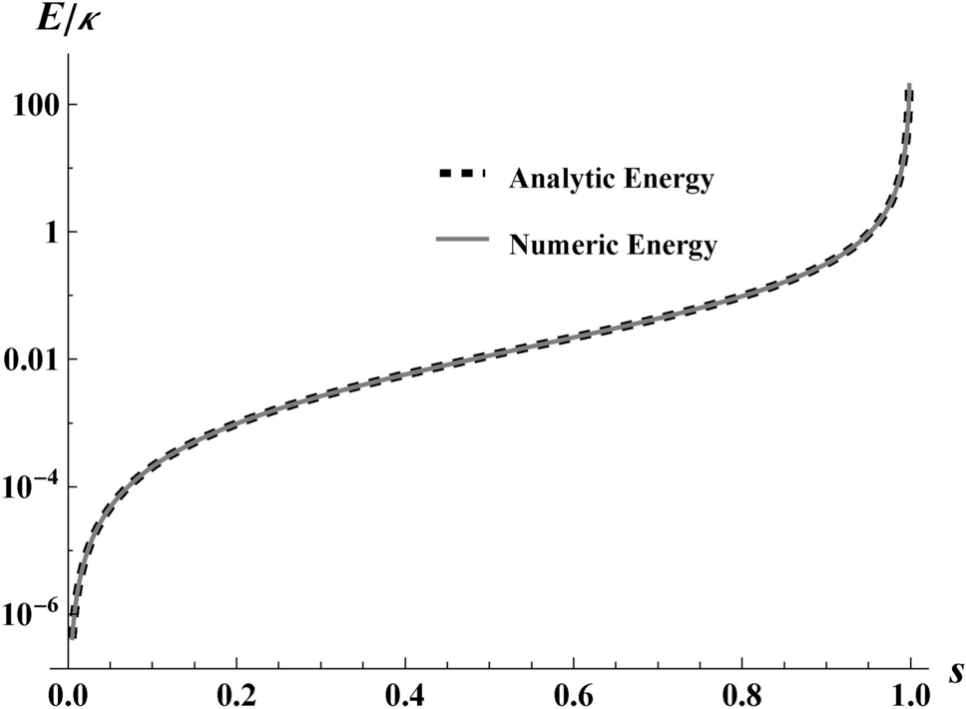
A plot of total energy, $E/\kappa$ as a function of maximum speed, *s* for $r=1$. The illustration helps demonstrate the equivalency of the analytic expression Eq. (9) (dotted black) with the numerical result of Eq. (21) (gray). A key physical takeaway is that a larger maximum speed exponentially increases energy production.

The limits of the integral are due to the finite displacement of the trajectory of our electron coming to rest at the edges of the box. We substitute $\textrm{d}{t}/\!\textrm{d}{x}$ by Eq. (3), and analytically integrate over the space from $x=-2r/\kappa$ to $x=+2r/\kappa$. The total energy is only a function of *r* and *s* (size and maximum speed). A plot of the result, Eq. (9), is given in Fig. 6 with fixed $r=1$, depicting finite emission energy as a function of *s*. It illustrates the energy divergence as $s\rightarrow 1$.

Despite its lengthiness, we express the energy *E*(*r*, *s*), Eq. (8), and its dependence on the box size, *r*, and maximum electron speed, *s*, for generality. Using $\gamma _s = 1/\sqrt{1-s^2}$, the answer is:

9 $$\begin{aligned} E(r,s) = \frac{e^2\kappa }{12\pi }\left( \frac{ s \gamma _s^2}{4 r} - 1 + E_+(r,s) + E_-(r,s)\right) , \end{aligned}$$

where $E_\pm$ are defined by,

$$\begin{aligned} E_\pm = A_\pm \tanh ^{-1}\Phi _\pm ^{-1/2},\quad \Phi _\pm = \frac{r(s\mp 1)}{r(s\mp 1)\pm s}, \end{aligned}$$

and $A_\pm$, $B_\pm$, $C_\pm$ are as follows:

10 $$\begin{aligned} A_\pm = B_\pm \left( 2+\frac{3 s^2}{8r^2} - 2\frac{s}{r} +C_\pm \right) , \nonumber \\ B_\pm =\frac{\Phi _\pm ^{1/2}}{(s\mp 1)^2},\nonumber \\ C_\pm = \pm \frac{5}{4 r} \mp \frac{1}{s} \mp s \mp \frac{s}{4r^2} \pm \frac{3}{4}\frac{s^2}{r}. \end{aligned}$$

When the electron is non-relativistic, Eq. (9), to leading order is

11 $$\begin{aligned} E(s) = \frac{e^2\kappa s^2}{36\pi }. \end{aligned}$$

We will prove this is the radiated amount of thermal energy released (only valid in the non-relativistic regime). But first, let us look at the limit of large displacements. For large distances traveled, $r\rightarrow \infty$ limit, the energy *E*(*r*, *s*) of Eq. (9) collapses to the following expression, in terms of final rapidity $\eta = \tanh ^{-1} s$:

12 $$\begin{aligned} \lim _{r\rightarrow \infty }E(r,s)=\frac{e^2\kappa }{12\pi }\left( \frac{\eta }{s}-1\right) . \end{aligned}$$

**Fig. 7:**
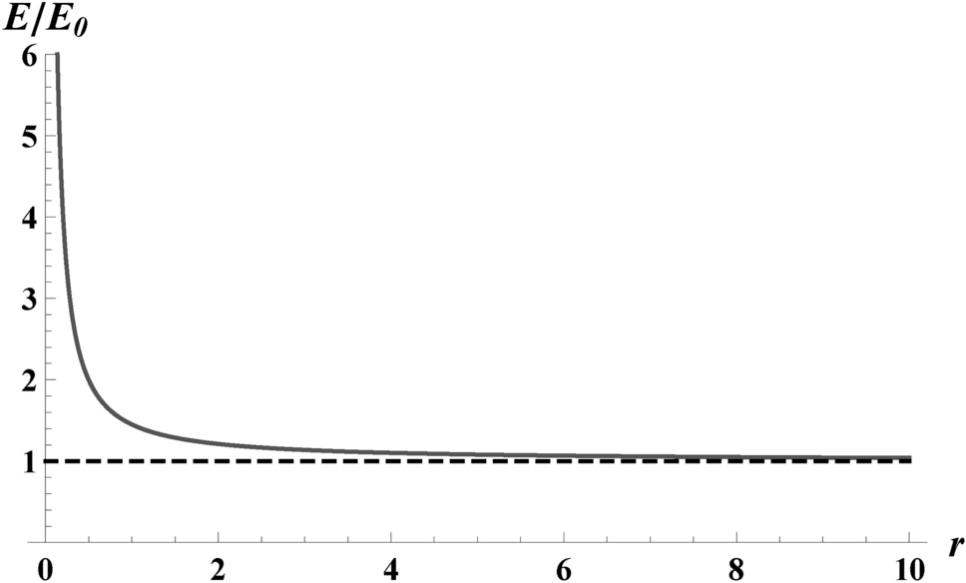
Total energy as a function of the distance traveled, *r*, by plotting Eq. (9) with $\kappa$; ${E}/{\kappa }$. The black dashed line shows the asymptotic behavior of the energy followed to large *r*. For visual clarity, the energy has been normalized by the minimum energy $E_0 = (\eta /s -1)\kappa e^2/12\pi$, from Eq. (12), which is the energy in the large *r* limit for $s=1/4$. The key takeaway is that the energy does not asymptotically drop to zero but instead approaches a finite value, Eq. (12). This is a signature of thermality.

The result shows a finite energy emission for the electron in the large distance limit; see Fig. 7. This is a critical result of this paper. This energy, Eq. (12), has the same dependence on final speed (not maximum speed) as the electron created during beta decay^14,31–33^.

Because beta decay has recently been shown to be thermal^13^ and measured^15,34^ as such, Eq. (12) suggests the radiation from Eq. (1) may be thermal in the large displacement limit, i.e., large boxes may contain CATs. However, to convincingly demonstrate a CAT, we must show that the spectrum has a Planck factor. We compute the spectrum for a particular box size in the following section, Sect. Spectral analysis, and specialize to small maximum speeds in Sect. Classical acceleration temperature to look for the Planck factor.

## Spectral analysis

### Spectral distribution, d*I*(ω)/dΩ

The spectral distribution for the straight-line traveling electron is calculated using the formula, see e.g., Eq. (14.70) of Ref^33^ or Eq. (23.89) of Zangwill^32^:

13 $$\begin{aligned} \frac{\!\textrm{d}{I(\omega )}}{\!\textrm{d}{\Omega }} =\frac{\omega ^{2}}{16\pi ^{3}}\sin ^{2}\theta \left| j(\omega ,k_{x})\right| ^{2}, \end{aligned}$$

where $j(\omega ,k_{x})$ is the current and $k_x=\omega \,\cos (\theta )$. The quantity $j(\omega ,k_{x})$ can be computed by:

14 $$\begin{aligned} j(\omega ,k_{x})=e\int \limits _{-2r/\kappa }^{2r/\kappa }e^{i\phi } dx, \end{aligned}$$

where $\phi =k_{x}x-\omega t(x)$. Then using Eq. (1), we get:

15 $$\begin{aligned} \phi /\omega =x \cos \theta -x\left( \frac{1}{s}-\frac{1}{r}\right) -\frac{2}{\kappa }\tanh ^{-1}\left( \frac{\kappa x}{2r}\right) , \end{aligned}$$

where we have substituted in expression $k_x=\omega \cos \theta$. The integral in Eq. (14) with $r=1$ can be performed with a substitution. Finally, complex conjugating the current gives:

16 $$\begin{aligned} \left| j(\omega ,k_x)\right| ^{2}= \frac{16\pi ^2 e^2 \omega ^2}{\kappa ^{4}} \textrm{csch}^{2}\left( \frac{\pi \omega }{\kappa }\right) \left| M\right| ^{2}, \end{aligned}$$

where *M* is the hyper-geometric Kummar function, $_1F_1$, dependent on *s*, the max speed of the electron, $\theta$ the polar angle, and $\omega$ the photon frequency. Explicitly,

17 $$\begin{aligned} M = _1F_1\left( 1-\frac{i \omega }{\kappa }, 2, \frac{4 i \omega }{\kappa }(1- \frac{1}{s} + \cos \theta ) \right) . \end{aligned}$$

The spectral distribution of Eq. (13) is, therefore,

18 $$\begin{aligned} \frac{\!\textrm{d}{I(\omega )}}{\!\textrm{d}{\Omega }}=\frac{e^2 \omega ^{4}}{\pi \kappa ^{4}} \sin ^{2} \theta \textrm{csch}^2 \left( \frac{\pi \omega }{\kappa }\right) \left| M\right| ^{2}. \end{aligned}$$

The spectral distribution, Eq. (18), which has set $r=1$, is a good starting point for investigating thermality, which we do in Sect. Classical acceleration temperature for low maximum speeds. However, in the following subsection, we will first integrate over the solid angle to get the $I(\omega )$ spectrum relevant for all maximum speeds $0<s<1$.

### Spectrum, *I*(*ω*)

We obtain the spectrum $I(\omega )$ by integrating over the solid angle, $\textrm{d}{\Omega }=\sin \theta \!\textrm{d}{\theta } \!\textrm{d}{\phi }$. The general prescription uses the current by integrating Eq. (13) employing Eq. (16):

19 $$\begin{aligned} I(\omega )=\frac{2 e^2 \omega ^{4}}{\kappa ^{4}}\textrm{csch}^{2}\left( \frac{\pi \omega }{\kappa }\right) \int \limits _{0}^{\pi }\sin ^{3}\theta \left| M\right| ^{2}d\theta . \end{aligned}$$

**Fig. 8:**
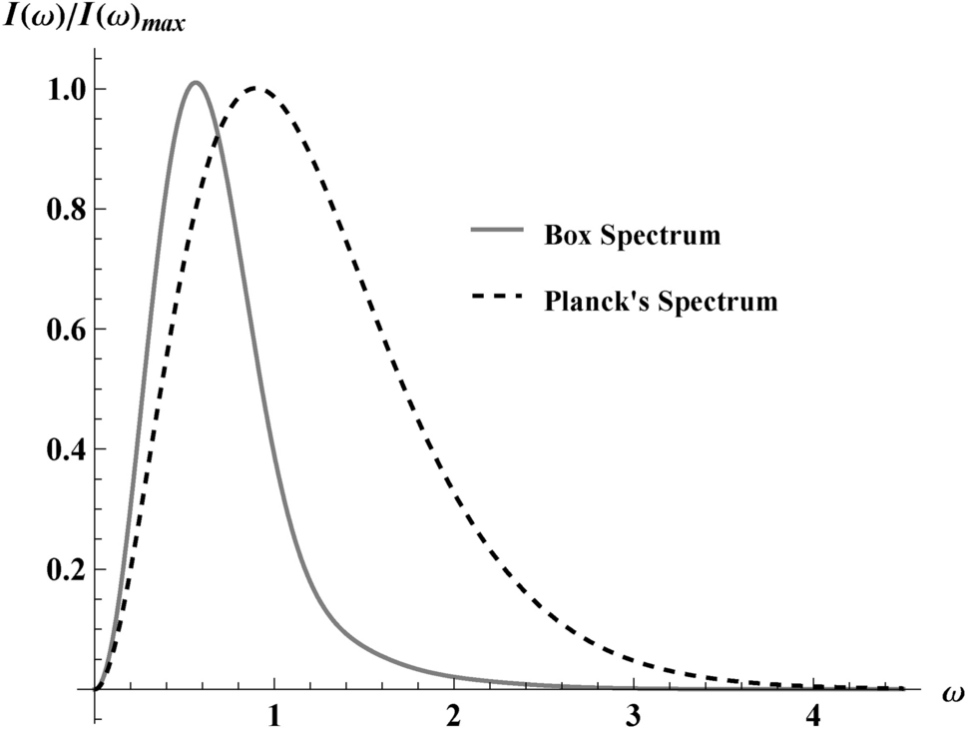
A plot of the spectrum, $I(\omega )$ vs $\omega /\kappa$ trend obtained from Eq. (19) for $s=0.4$ asymptotically approaching 0 as $\omega \rightarrow \infty$. The spectrum is compared to the 3+1 Planck’s spectrum (black dashes), $I_{\text {Planck}}(\omega ) =\omega ^{3}\left( e^{\frac{2\pi \omega }{\kappa }}-1\right)$. The figure compares the box spectrum, Eq. (19), for the trajectory, Eq. (1) with the standard $3+1$ dimensional Planck spectrum. The box spectrum shares the same basic asymptotic profile as Planck. The plots are normalized by their maximum values $I_{\text {max}}=(0.0458,0.0078)$ for the Planck and box, respectively.

In general cases, the $I(\omega )$ spectrum is not normally analytic, and Eq. (19) is no exception. However, as we will show, an analytic form for the spectrum exists at non-relativistic speeds, Eq. (31). We have plotted the spectrum $I(\omega )$, Eq. (19) in Fig. 8. Notice the lack of an infrared divergence, which is expected for an asymptotically static trajectory, unlike asymptotic constant velocity trajectories like the beta decay trajectory, see, e.g., Ref^15,35^.

### Energy from spectrum, *E*(*s*)

To compute the total energy of the classical radiation emitted by the electron, $E_{\text {total}}$, one integrates Eq. (13) over the solid angle, $\textrm{d}{\Omega }$ and frequency, $\textrm{d}{\omega }$ via:

20 $$\begin{aligned} E_{\text {total}}=\int \limits _0^\infty \frac{\textrm{d}{I(\omega )}}{\textrm{d}{\Omega }} \textrm{d}{\Omega }\textrm{d}{\omega } =\int \limits _{0}^{\infty }I(\omega ) \textrm{d}{\omega }, \end{aligned}$$

Using the spectra $I(\omega )$ from Eq. (19), we obtain energy emitted, remembering that $r=1$, as:

21 $$\begin{aligned} E_{\text {total}}^{r=1}(s)=\int \limits _{0}^{\infty }\int \limits _{0}^{\pi }\frac{2 e^2 \omega ^{4}}{\kappa ^{4}}\textrm{csch}^2\left( \frac{\pi \omega }{\kappa }\right) \sin ^{3}\theta \left| M\right| ^{2} d\theta d\omega . \end{aligned}$$

Numerical integration of the above equation gives $E_\text{total}$ as a function of max speed, *s*. The total energy of Eq. (21) checks with the energy Eq. (9) obtained from the Larmor power, verifying consistency and the correctness of the $r=1$ spectral distribution Eq. (18). The equivalency of equations Eqs. (21) and (9) is illustrated in Fig. 6.

## Classical acceleration temperature

This section will analyze the spectral distribution, Eq. (18), (which assumes box size $r=1$) and demonstrate thermality in the non-relativistic regime, $s\ll 1$. However, first, we will look at the character of the acceleration in the relevant limits to understand and motivate the situation from a temporal-spatial perspective.

### Peel acceleration, $|\mathcal {P}| = \kappa$

A lesser-known yet significant thermal object associated with acceleration, referred to as the ‘peel,’ e.g., Ref^16,36–38^, defined with null coordinates as $V''(U)/V'(U)$, has garnered interest due to its implications in understanding thermal phenomena with time-dependent motion. This section demonstrates that the peel remains constant to first order in large box sizes or low speeds. This result hints at thermality and suggests Eq. (18), valid for $r=1$, and possessing no apparent Planck factor, may be thermal in the regime where speeds are low, i.e. $s\ll 1$.

Consider the Carlitz–Willey trajectory^36^:

22 $$\begin{aligned} V = \frac{1}{\kappa } e^{\kappa U},\qquad U = t - x. \end{aligned}$$

This expression is written in light-cone coordinates where $V= t+x$. One can use light-cone coordinates, (*V*, *U*), and express the peel of the above Carlitz-Willey trajectory in a simple way. The constant peel gives the temperature of the quantum radiation from the moving mirror:

23 $$\begin{aligned} \kappa = \frac{V''(U)}{V'(U)},\qquad T_{\text {mirror}} = \frac{1}{2\pi } \frac{V''(U)}{V'(U)} = \frac{\kappa }{2\pi }. \end{aligned}$$

Create a similar peel by considering the following equation of motion for a moving electron in the context of beta decay^15^,

24 $$\begin{aligned} x = \frac{s}{\kappa } e^{\kappa u_s},\qquad u_s = t - \frac{x}{s}, \end{aligned}$$

where *s* is the maximum speed of the electron. This trajectory resembles the eternal thermal Carlitz-Willey moving mirror in the sense that the peel in the coordinates $(x,u_s)$ is constant, which gives the temperature:

25 $$\begin{aligned} \kappa = \frac{x''(u_s)}{x'(u_s)}, \qquad T_{\text {electron}} = \frac{1 }{2\pi } \frac{x''(u_s)}{x'(u_s)} = \frac{\kappa }{2\pi }. \end{aligned}$$

Notice that $u_s$ is not the usual light-cone coordinate but a ‘stretched-space’ retarded time coordinate.

With an expression for the peel in terms of stretched-space in hand, let us compute the first expression of Eqs. (25) for our boxed trajectory, Eq. (1),

26 $$\begin{aligned} \mathcal {P} \equiv \frac{x''(u_s)}{x'(u_s)} =\pm \kappa \left[ 1+\frac{1}{r}\left( \frac{1}{v}-\frac{1}{s}\right) ^{-1}\right] ^{3/2}, \end{aligned}$$

where *v* is the velocity, Eq. (4), of the trajectory Eq. (1). It is readily seen that for large box sizes *r*,

27 $$\begin{aligned} \mathcal {P} = \pm \kappa + \mathcal {O}(1/r). \end{aligned}$$

Like the energy result, Eq. (12), obtained in the limit of large *r*, the dynamic (temporal-spatial) result, Eq. (27), also suggests thermality is present for large *r*. It should be noted that for small velocities $s \rightarrow 0$, Eq. (26) gives

28 $$\begin{aligned} \mathcal {P} = \pm \kappa + \mathcal {O}(s), \end{aligned}$$

which suggests thermality is present for non-relativistic speeds. On the other hand, for relativistic motion, the peel is not a constant $\kappa$ at the next-to-leading order in *s*, i.e., $\mathcal {P} = \pm \kappa \mp \frac{3 \kappa s}{2 r} + \mathcal {O}(s^2)$. We consider a constant peel a necessary, though not sufficient, condition for a particle spectrum to qualify as a CAT. Numerical analysis shows that a non-constant peel is commensurate with deviations from a strict thermal spectrum, confirming that the overall relativistic spectrum is not Planckian. Therefore, in the relativistic regime, the spectrum will not meet the criteria for CAT, even though higher-order corrections may contain Planck-like terms.

In summary, our trajectory, Eq. (1), in the limit of $r \rightarrow \infty$ or $s\ll 1$, may exhibit thermality as given by the temperature, $T=\kappa /2\pi$, which is a conjecture by analogy with the temperatures of the moving mirror trajectory Eq. (22), and the electron trajectory of beta decay, Eq. (24).

### Proper acceleration, *α*(*v*)

The proper acceleration, Eq. (5), of trajectory Eq. (1) is identical to the thermal acceleration exhibited during beta decay, $\alpha _0(v) = \kappa v \gamma ^3(1-v/s)^2$^14^, in the leading order for a large box. This is consistent, as it should be, with the total energy emitted shown in Eq. (12), which assumed a large finite displacement.

It is straightforward to see the leading order of Eq. (5) in terms of large *r*,

29 $$\begin{aligned} \alpha (v) = \alpha _0(v) + \mathcal {O}(1/r). \end{aligned}$$

In addition to expanding in terms of large *r*, one may also expand to leading order in small speeds *s*,

30 $$\begin{aligned} \alpha (v) = \frac{\kappa v^3 \gamma ^3}{s^2} + \mathcal {O}(1/s). \end{aligned}$$

This result is identical to low speeds, $s\ll 1$, of beta decay acceleration, $\alpha _0(v) = \kappa v \gamma ^3(1-v/s)^2 \approx \kappa v^3 \gamma ^3 / s^{2}$, which suggests an electron moving along Eq. (1) in the non-relativistic regime, emits thermal radiation. Taken together, Eqs. (28) and (30), properly motivate a study of the spectrum. At low speeds, one expects a Planck distribution, as given by Eq. (31), which allows us to identify a temperature, Eq. (36).

### Planck’s law, *r* = 1 & *s*$\ll$ 1

The spectral distribution, Eq. (18), assumes box size $r=1$ and may demonstrate thermality in the non-relativistic regime, $s\ll 1$. At these low speeds, we can take the spectral distribution to leading order in $s^2$ and integrate over the solid angle to get the spectrum:

31 $$\begin{aligned} I_{\text {NR}}(\omega ) = \frac{e^2 s^2 }{3 \pi ^2} \frac{2\pi \omega /\kappa }{e^{2 \pi \omega /\kappa } -1} + \text {O.T.'s}. \end{aligned}$$

The oscillation terms, $\text {O.T.'s} = X Y$, the product of a complicated function

32 $$\begin{aligned} X(\omega ) = \frac{\textrm{csch}^2\pi \omega /\kappa }{32e^{4\pi \omega /\kappa }}\left[ \frac{\sin {(4\omega /\kappa )}}{4\omega /\kappa }-\cos {(4\omega /\kappa )}\right] e^{2\pi \omega /\kappa }. \end{aligned}$$

and another, also complicated, *s*-dependent function,

33 $$\begin{aligned} Y(\omega ,s) = -\frac{2^{\frac{4 i \omega }{\kappa }} e^{-\frac{4 i \omega }{\kappa }}}{\Gamma \left( \frac{i \omega }{\kappa }+1\right) ^2}s^2 e^{\frac{4 i \omega }{\kappa s }} \left( -\frac{i\omega }{ \kappa s}\right) ^{\frac{2 i \omega }{\kappa }} + \text {h.c.} \end{aligned}$$

An integral of $I_{\text {NR}}(\omega )$, Eq. (31), however, gives a surprisingly clean result:

34 $$\begin{aligned} E = \int \limits _0^\infty I_{\text {NR}}(\omega ) \!\textrm{d}{\omega } = \frac{e^2\kappa s^2}{36\pi }, \end{aligned}$$

which agrees with Eq. (11), confirming the form of the spectrum, Eq. (31).

**Fig. 9:**
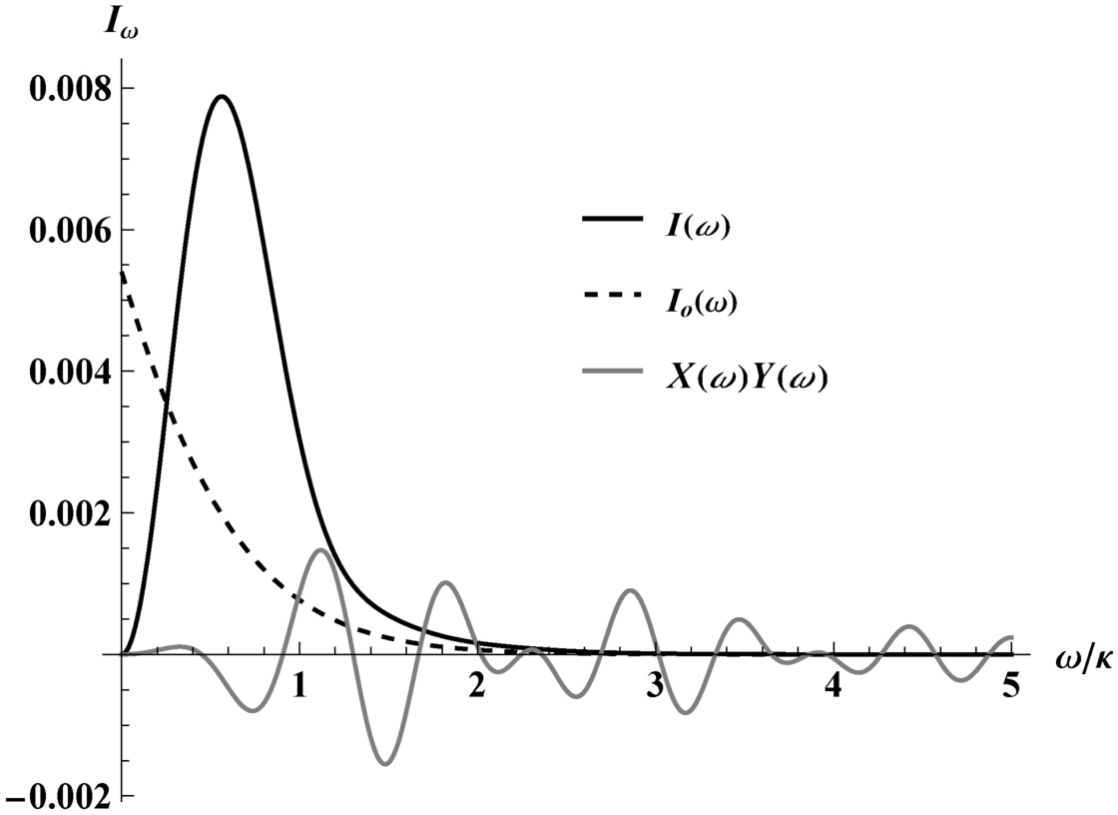
The figure shows that the oscillations of the energy spectrum do not contribute to the energy emission. $I_\omega$ ($\kappa =1$) represents the different spectra labeled as $I(\omega )$, $I_0(\omega )$, and $X(\omega )Y(\omega )$; that is, the relativistic energy spectrum Eq. (19), the non-relativistic leading order term Eq. (35), and the oscillatory terms of Eq. (31), respectively. Here $s=0.4$.

Interestingly, neglecting the oscillation terms responsible for IR-finite regularization is possible in the above integral, as they do not spoil the Planck distribution; see a similar situation in Ref^16^. That is, we can use the first term in the spectrum Eq. (31), named $I_0(\omega )$, to show that:

35 $$\begin{aligned} I_0(\omega ) = \frac{e^2s^2 }{3 \pi ^2} \frac{2\pi \omega /\kappa }{e^{2 \pi \omega /\kappa } -1}, \quad E = \int \limits _0^\infty I_0(\omega ) \!\textrm{d}{\omega } = \frac{e^2\kappa s^2}{36\pi }, \end{aligned}$$

demonstrating that the oscillation terms do not contribute to the total radiation energy. As an illustration of the non-relativistic spectrum, $I_{\text {NR}}(\omega )$, from Eq. (31), we have plotted the first term, $I_0(\omega )$, and compared it with the oscillatory terms, $X(\omega )Y(\omega )$, as well as the fully relativistic spectrum, $I(\omega )$, from Eq. (19) in Fig. 9. It is seen that the area under the oscillation curve is zero and thus does not contribute to the energy emission.

The temperature in $I_0(\omega )$ of Eq. (35), reinstating Kelvin SI units,

36 $$\begin{aligned} T = \frac{\hbar \kappa }{2\pi c k_B}, \end{aligned}$$

is independent of max speed *s* or polar angle $\theta$. This is in contrast to other cases like the asymptotic static trajectory for complete evaporation associated with the Schwarzschild–Planck system in Ref^16^, the remnant trajectories in Ref^35^, or the eternal black hole trajectory in the appendix of Ref^13^. Notice that $\hbar$ is required for all temperatures measured in Kelvin^39^. Its appearance in this classical computation does not signify any use of quantum theory, i.e., Eq. (36) is a CAT.

## Particle production

### Particle spectrum, *N*(*ω*)

We can compute the particle spectrum $N(\omega )$, noticing the quantum inclusion of $\hbar$, by using the following relation:

37 $$\begin{aligned} N(\omega ) = \frac{I(\omega )}{\hbar \omega }. \end{aligned}$$

That is, the particle spectrum can be calculated as an integral of the spectral distribution:

38 $$\begin{aligned} N(\omega )=\int \limits _{0}^{\pi }\int \limits _{0}^{2\pi }\frac{1}{\hbar \omega }\frac{\!\textrm{d}{I}}{\!\textrm{d}{\Omega }}\sin \theta \!\textrm{d}{\theta } \!\textrm{d}{\phi }. \end{aligned}$$

This assumes each particle carries a quantum of energy $\hbar \omega$, where the idea of photons in classical electrodynamics is considered semi-classical^33^.

### Fixed box size, *r* = 1

We specialize to box size $r=1$ and use Eq. (18) in Eq. (38), integrate over $\phi$, and obtain the particle spectrum $N(\omega )$ as a function of frequency $\omega$,

39 $$\begin{aligned} N(\omega )=\int \limits _{0}^{\pi } \frac{2 e^2 \omega ^{3}}{\hbar \kappa ^{4}}\textrm{csch}^2(\frac{\pi \omega }{\kappa })\sin ^{3}\theta |M|^{2} \!\textrm{d}{\theta }. \end{aligned}$$

**Fig. 10:**
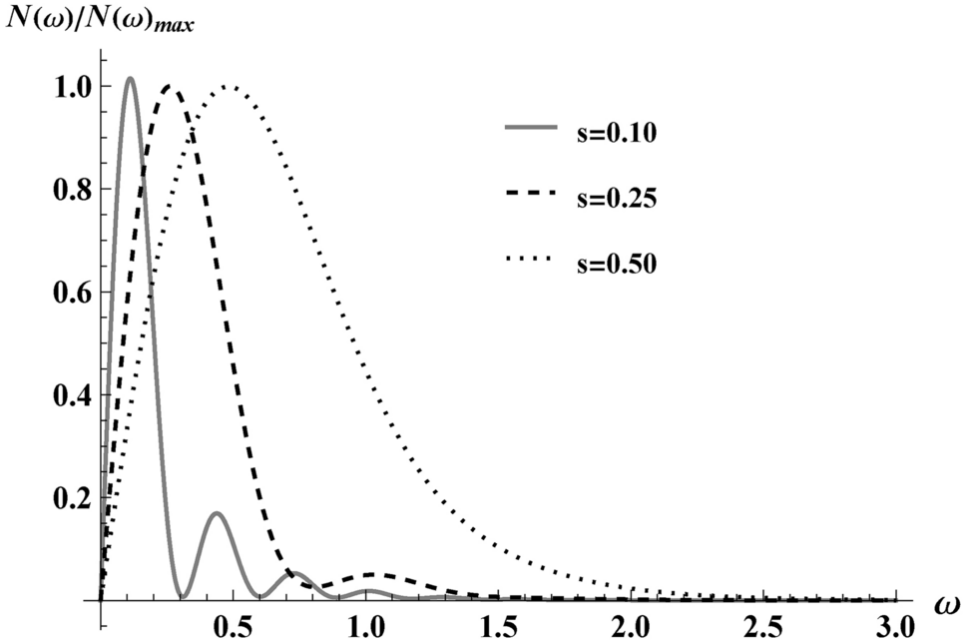
$N(\omega )/N(\omega )_{\text {max}}$ vs $\omega /\kappa$ maximum speeds, $s\rightarrow (0.10,0.25,0. 50)$ via the numerical solution of Eq. (39), at $r=1$. Here $N(\omega )$ is normalized to its peak values: $N_{0.1}=0.46\times 10^{-2}$, $N_{0.25}=1.09\times 10^{-2}$ and $N_{0.50 }=1.96\times 10^{-2}$, respectively. Notice the appearance of a tail oscillation due to lower maximum speed (dashed line). These oscillations are critical for regularizing finite particle count but do not contribute to the radiated energy.

This integral cannot be done analytically. Nevertheless, numerically evaluating Eq. (39), we obtain the particle spectrum in Fig. 10. The fact that the worldline comes to a stop is the dynamic reason the particle spectrum $N(\omega )$ suffers no IR-divergence, see e.g. Ref^13–15^. To better understand Eq. (39), we need to specialize to non-relativistic speeds, which we do in the next section.

### Non-relativistic max speed, *r* = 1 & *s*$\ll$ 1

Low maximum speeds are characterized by $s\ll 1$ where we still have $r=1$. Eq. (37) is succinctly reformulated using the $I_0(\omega )$ in Eq. (35) as follows:

40 $$\begin{aligned} N_0(\omega )= \frac{2}{3\pi }\frac{e^2 s^2}{\hbar \kappa } \frac{1}{e^{2\pi \omega /\kappa }-1}. \end{aligned}$$

We have neglected the oscillation terms because we have used Eq. (35) and not the full expression Eq. (31) here, but as can be seen, they are precisely the needed ingredients to regularize the infrared divergence. In contrast to energy, the finite total particle count depends on the oscillations. This is because

41 $$\begin{aligned} N_0(\omega ) = \frac{I_0(\omega )}{\hbar \omega },\quad N_\infty = \int \limits _0^\infty N_0(\omega ) \!\textrm{d}{\omega }, \end{aligned}$$

is not finite (IR-divergent), and $N_\infty$ does not count the particles. Instead, the total particle count

42 $$\begin{aligned} N = \int \limits _0^\infty N(\omega ) \!\textrm{d}{\omega } = \text {finite}, \end{aligned}$$

is convergent and includes the oscillations, via $N(\omega )$ of Eq. (37). The fact that the energy *E*, Eq. (35), has no dependence on the oscillation terms and the particle numbers do, Eq. (42), can mean two different things physically: (a) the oscillation part produces soft particles that do not carry energy (These O.T.’s might rightly characterize infinite ‘anti-soft’ particles that regularize the infinite soft particles of the 1D Planck term.), or (b) some particles produced in the oscillations produce positive energy while some negative energy, and they exactly cancel. In Ref^40^, particle numbers do not reveal negative energy flux; thus, it is an open question on how to physically interpret the particle production associated with the oscillations .

The thermality observed using the trajectory Eq. (1) arises from a spectrum combining a 1+1 dimensional Planck distribution with oscillatory terms, effectively transforming the overall result into a 3+1 dimensional spectrum. The oscillatory terms can be analogously interpreted as effective backscattering and graybody factors associated with black hole radiance, e.g., Ref^41^. They do not contribute to the total energy radiated but are essential for regularizing the infrared regime to yield a finite photon number.

**Fig. 11:**
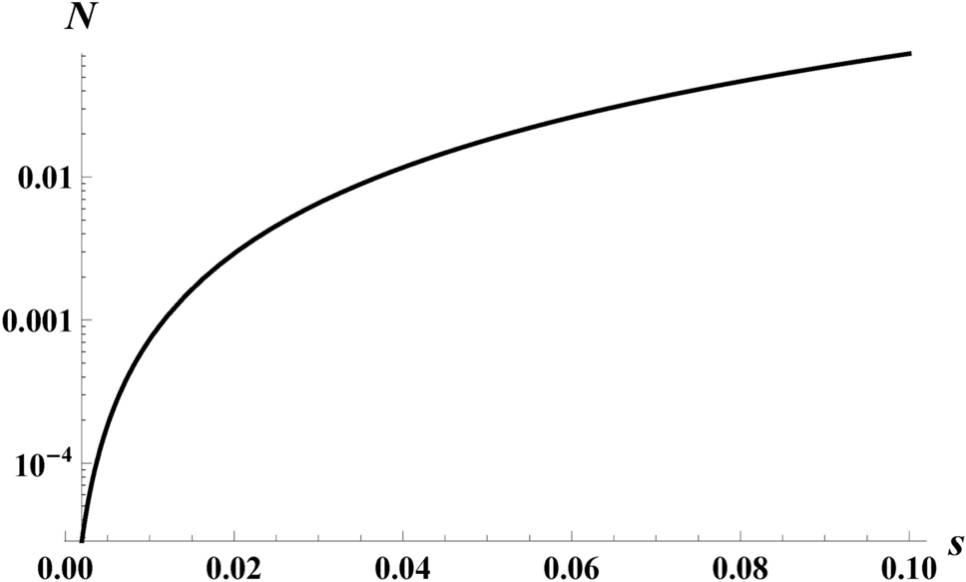
Particle number, *N*, as a function of *s* in the non-relativistic regime $s\rightarrow (0,0.1)$, shows monotonic increase in approach to the relativistic regime for $r=1$.

The particle count results of Eq. (42) using Eq. (37) via Eq. (31) with the oscillation terms, Eqs. (32) & (33), in the non-relativistic regime are plotted in Fig. 11. Notice the faster the maximum speed of the electron, the more particle creation.

Having derived the photons, let us consider the scalars from the dual mirror moving along Eq. (1). The quantum radiation emitted by an accelerating mirror to the right is found using the mirror-electron mapping recipe see, e.g., Ref^13,42^. One finds from Eq. (18) with $s\ll 1$ and $r=1$, the scalar particle production beta Bogolybov coefficients,

43 $$\begin{aligned} |\beta _{pq}|^2 =\frac{4 s^2}{\kappa \pi } \frac{p q }{(p+q)^3} \frac{1}{e^{2\pi (p+q)/\kappa }-1}, \end{aligned}$$

where we have ignored the oscillation terms. The scalars (not photons) carry energy emitted to both sides of the mirror, analog-consistent with Eq. (11), (see, e.g., the general form via Eq. (43) of Ref^16^),

44 $$\begin{aligned} E_{\text {mirror}} = \int \limits _0^\infty \int \limits _0^\infty \hbar (p+q) |\beta _{pq}|^2 \!\textrm{d}{p}\!\textrm{d}{q} = \frac{\hbar \kappa s^2}{36\pi }. \end{aligned}$$

We emphasize that the moving mirror in (1+1)-dimensions is *not* an actual electron in (3+1)-dimensions, though, there is an exact, functional, identity mapping between the two. The point is that the scalar particle spectrum emitted by the (1+1) moving mirror has a one-to-one photon particle spectrum counterpart emitted by the (3+1) ordinary electron. In other words, there is a “duality” between the two systems^10^, a classical-quantum correspondence whose physical content in 3d space is encoded on a line. In the high-frequency limit^1^, $p\gg q$, Eq. (43) gives a result similar to Hawking radiation for particle production,

45 $$\begin{aligned} |\beta _{pq}|^2 = \frac{4 s^2}{\kappa \pi }\frac{q}{p^2} \frac{1}{e^{2\pi p/\kappa }-1}, \end{aligned}$$

where the Planck factor demonstrates $T=\hbar \kappa /2\pi c k_\text {B}$ thermality in frequency *p*, in analog to the late-time Schwarzschild black hole^43^; see also e.g. the late-time Schwarzschild mirror^44^ via Eq. (B3) of Ref^13^, Eq. 3.24 of Carlitz-Willey^36^, or Eq. (1) of Fulling^45^. Equation (45)’s thermality characterizes the quantum radiation emitted by the moving mirror and establishes the non-relativistic electron along Eq. (1) as a thermal analog of black hole evaporation.

## Conclusions

We have found that a confined, non-periodic, slowly moving, straight-line accelerating electron emits thermal radiation. The key traits are finite particles and energy emission from a finite space. It is experimentally favorable that the thermal effect from the bounded region coincides with the non-relativistic regime. For clarity and computational traction, the travel distance for non-relativistic motion along its specific worldline is described by the variable *r*, which only yields analytic results [e.g., the spectrum Eq. (31)] when $r=1$. In this case, the total distance traveled is finite, which is favorable for finite-sized laboratories.

Furthermore, *r* emerges as a crucial factor in determining the dimensional characteristics of the spectrum under investigation. Our study reveals that as *r* increases towards infinity, the spectrum transitions towards 1+1 dimensional behavior. At the same time, $r=1$ yields a particle spectrum with no IR-divergence similar to a 3+1 dimensional Planck spectrum. This finding shows that *r* can play a role in calibrating analog grey body factors as is known in black hole radiance, effectively capturing the influence of backscattering and the particle spectrum dimensionality. Novel physical results include:

- Total energy radiated depends on the finite distance traversed; see Eq. (9).
- Total energy radiated does not depend on the finite distance traversed for large travel distances, see Eq. (12).
- For non-relativistic speeds (and fixed box size), the photons are Planck-distributed, Eq. (31).

The total energy, Eq. (9), was found analytically in its most general form and is not straightforward. Nevertheless, the simple result is that the amount of energy radiated does not change with distance when the distance is large, Eq. (12). The result scales as the total energy radiated by the unconfined electron emitting thermal photons during beta decay. Therefore, in the context of Eq. (1), it is natural to conjecture that large boxes have CATs. Still, a Planck distribution cannot be confirmed from the seemingly intractable form of the large-box spectrum. Regardless, for a fixed travel distance, the non-relativistic moving electron emits Planck-distributed photons; thus, the radiation is demonstrably thermal, Eq. (36). There is a CAT in the box.

## Data Availability

All data generated or analyzed during this study are included in this manuscript.
